# Advanced method for fabrication of molecularly imprinted mesoporous organosilica with highly sensitive and selective recognition of glyphosate

**DOI:** 10.1038/s41598-019-46881-7

**Published:** 2019-07-16

**Authors:** Youngdo Kim, Jaeho Lee, Ik-Soo Shin

**Affiliations:** 1 Biosensor and Materials Group, Korea Institute of Science and Technology Europe, Universität des Saarlandes Campus E7 1, 66123 Saarbrücken, Germany; 2 Department of Chemistry, College of Natural Science, Soongsil University, Seoul, 06978 Republic of Korea

**Keywords:** Predictive markers, Nanoparticles

## Abstract

In this study, we synthesized molecularly imprinted mesoporous organosilica (MIMO) in the presence of a new precursor having a zwitterionic functional group and an imprint molecule, namely, glyphosate (MIMO-z). The precursor-glyphosate complex engaged in a typical base-catalyzed sol-gel reaction and the introduced zwitterion group remained intact in the framework after the extraction process had been completed. To test the rebinding performance of the target molecule, graphene quantum dots were encapsulated (MIMO-zQ) into pores and the fluorescence intensity change was monitored according to the concentration of glyphosate. When the MIMO-zQ suspension was diluted into the glyphosate solutions, notable fluorescence quenching occurred, right down to sub-nanomolar levels of concentration; 9.2 ± 0.18% quenching at 0.1 nM (0.017 ppb, 17 *p*g/mL). This result is one of the best reported to date for sensing using MIMO. The synthesized probe also exhibited a distinct signal compared to a series of competing compounds, aminomethylphosphonic acid and glycine; 4.3 ± 0.019% and 3.7 ± 0.041% quenching at 100 nM.

## Introduction

One of the most controversial nonselective herbicides which has potential carcinogenicity^1^ and endocrine disruption^2^ in humans is 2-(Phosphonomethylamine)acetic acid (glyphosate, Glyp). It is a systemic and postemergence organophosphorus herbicide, and is used extensively in agricultural, forestry, and aquatic applications^3^. Although its toxicity is relatively low to mammals, indiscriminate use of it can lead to high level doses in agricultural products, which in turn, can cause disorders in humans^4,5^. Large scale global consumption of this herbicide is contributing to its appearance in various environmental matrices such as water, sediment and soil^6^. Its residues have also been found in food, drinking water and groundwater, thereby resulting in positive findings of its presence in human urine and breast milk. In the EU, the maximum residue levels have already been established, for most plants and animal commodities; being limited to the quantification of 0.1 mg/kg and 0.05 mg/kg, respectively^7^. Therefore, early detection and assessment are essential elements in elucidating the impact on humans. The most widely used method is mass spectrometry, based on chromatographic analysis such as gas chromatography or liquid chromatography^8^. However, these methods are very expensive, require laboratory-scaled instruments and the employment of highly specialized technical personnel, along with a number of time-consuming stages in sample preparation. Since glyphosate is a small molecule of high polarity, and is known to be insoluble in organic matter, additional pre- or post-column processes are mandatory for the achievement of accurate results. Various direct detection methods which seek to overcome the demerits of chromatographic methods have increasingly been used and reported over the past decade. These include the following; enzyme-linked immunosorbent assay^9^, colorimetry^10–12^, fluorimetry^13–15^, chemiluminescence^16^, electrochemiluminescence^17,18^, electrochemical analysis^19–21^, especially when combined with many other selectivity enhancing approaches namely; self-assembly, surface modified Au nanoparticles, QDs, carbon nanotubes and MIPs. However, many challenges still exist in glyphosate analysis and more suitable and effective methods are urgently required^22^.

Since the mesoporous silica is synthesized using cationic surfactants with a sol-gel reaction, considerable efforts have been made to enhance its functionality in a wide variety of applications by the hybridization of organic species. Such applications include use a catalyst, for separation, drug delivery, optics, electronic devices, pollutant adsorption for environmental applications, as well as sensing^23,24^. Due to the formation of highly cross-linked silica matrix by sol-gel chemistry, homogeneous recognition sites can be generated through the process of molecular imprinting for selective sensing applications. However, the delicate cavities on the surface were not imprinted and formed to the degree sought by the simple addition of imprint molecule during the sol-gel reaction with the tetraalkyl silicates^25^. To overcome this difficulty, Davis’s and Chang’s group suggested that the imprint molecule derivative silane precursors ought to be introduced into the reaction process in order to be involved in the silica formation^26,27^. These were observed to have sacrificial carbamate bonds with the (3-aminopropyl)triethoxysilane which can be broken down through hydrolysis to remove only the imprint molecules. This approach was very successful in creating the imprinted cavities sought; however, one limitation is that it is only applicable to the hydroxyl group possessing compounds. Structurally, mesoporous organosilica provides a facile nanochannel pathway and this has been investigated in a variety of sensing applications^28^, especially when engrafted with molecular imprinting techniques^29,30^. This process works by promoting site formation recognition between the pores of the mesoporous organosilica networks. In addition, its high pore volume and nano-sized wall thickness allow for the introduction of quantum dots (QD) in the vicinity of the imprinted cavities located on the framework. Kim *et al*. detected bisphenol A at concentrations as low as 50 ng/mL, and was able to do so by monitoring QD quenching with MIMO^31^.

Herein, we report an alternative and relatively advanced glyphosate recognizing probe based on the molecularly imprinted mesoporous organosilica nanoparticles, which can be created by the simple addition of a glyphosate bound precursor containing zwitterionic moiety during synthesis. One major aim of this paper was to probe glyphosate selectively against structural analogues by the template-dependent nature of the molecular imprinting process, whilst at the same time incorporating the zwitterionic group of the precursor. Quantum dots encapsulated in pores are used as a signal transducer to detect levels of target sensitively by monitoring the fluorescence quenching behavior, as it responds to the concentration of analytes dissolved in de-ionized water.

## Results

**Figure 1:**
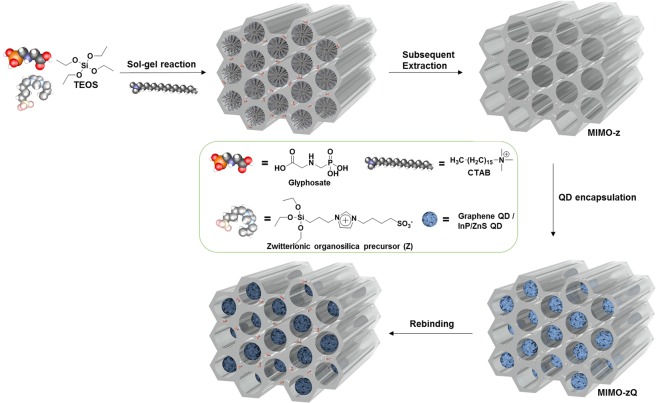
Schematic illustration of the synthesis of QD-encapsulated glyphosate imprinted mesoporous organosilica (MIMO-zQ).

As an initial design principle of a glyphosate-imprinted mesoporous organosilica (MIMO-z) to probe glyphosate, we first prepared a zwitterionic silica precursor which was designed to contain both cationic imidazolium and anionic sulfonate groups. This enables electrostatic interaction and hydrogen bonding with glyphosate. As illustrated in Fig. 1, the precursor-glyphosate complex participated in the sol-gel reaction together with tetraethyl silicate (TEOS) and a structure-directing surfactant, hexadecyl(trimethyl)azanium bromide (CTAB), to synthesize the glyphosate-embedded silica. In order to produce MIMO-z, both CTAB and glyphosate were removed simultaneously. The main IR absorption bands of the target-embedded silica at 3000–2800 and 1480 cm^−1^ which correspond to the long alkyl chains of CTAB^32^ and NH_2_ deformation of glyphosate^33^, respectively, were confirmed to have completely disappeared in the case of MIMO-z (Supplementary Fig. 1). This proves that both the mesoporous silica matrix and the glyphosate-specific imprinted cavity had been formed, and yielded MIMO-z as a result. Its pore surface was further functionalized using 11-trimethoxysilylundecane-1-thiol and we chose two kinds of quantum dots (QDs) for the encapsulation *via* the ligand exchange process (MIMO-zQ: graphene QD; MIMO-zQ’: InP/ZnS QD). Each QD has different emission wavelength and size, and thereby we confirmed that the size of QD relates to the recognition performance.

**Figure 2:**
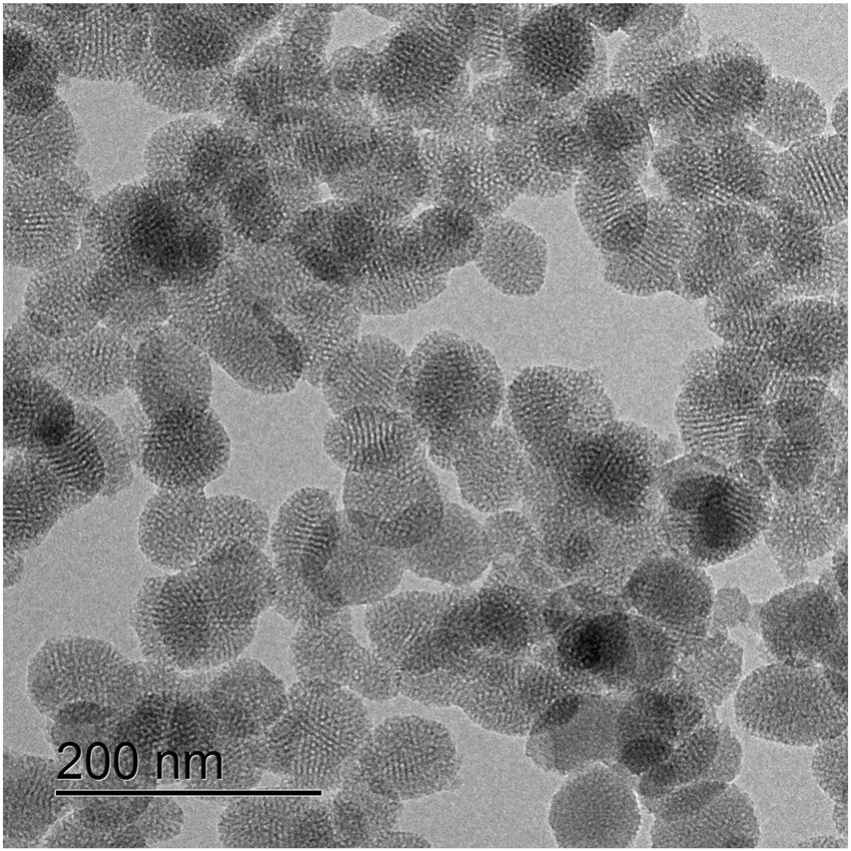
High resolution TEM micrograph of MIMO-z.

Several means of analyses, such as the Brunauer-Emmett-Teller (BET) surface area analysis, small angle X-ray scattering (SAXS), and ^13^C solid state NMR were used to reveal the structural information that is responsible for the sensing performance of MIMO-zQ’s (see Supplementary Notes 1 and 2). Figure 2 shows HRTEM images of the MIMO-z, highlighting the size and morphology of the nanoparticles. The average diameter falls within the range of 50 nm and a porous structure is clearly observed. InP/ZnS QDs were also observed to be densely concentrated at the pore surface of MIMO-z (Supplementary Fig. 3a). The inset image also indicates the retention of the size^34^ (~1.8 nm) and the lattice spacing of QDs (0.22 nm) (Supplementary Fig. 3b).

**Figure 3:**
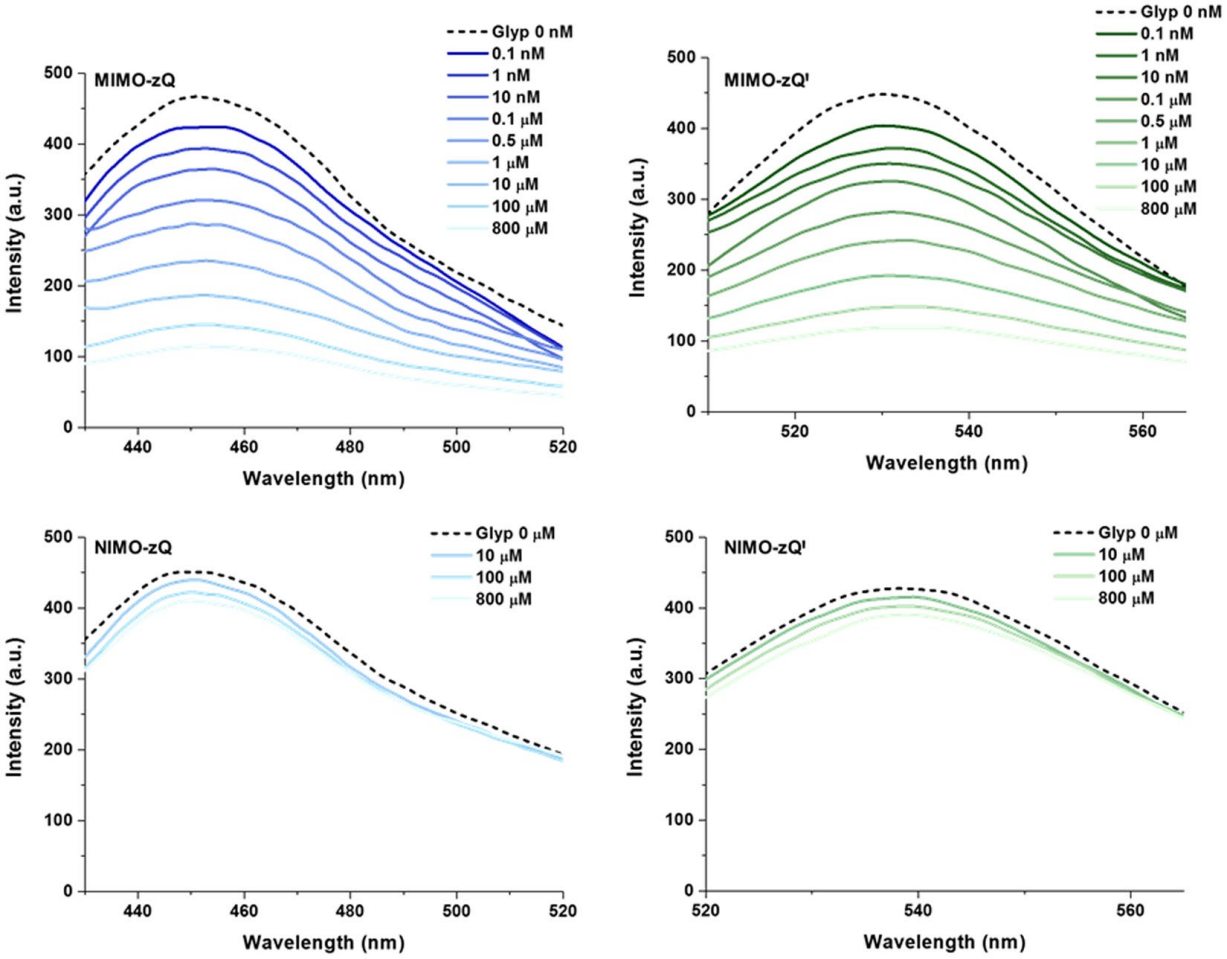
Photoluminescence (PL) emission as a function of the analyte concentration: PL spectra of graphene QD and InP/ZnS QD incorporated MIMO-z (MIMO-zQ and MIMO-zQ’). Also shown are the PL spectra of graphene QD and InP/ZnS QD incorporated NIMO-z (NIMO-zQ and NIMO-zQ’), which represent the absence of imprinted binding sites.

**Figure 4:**
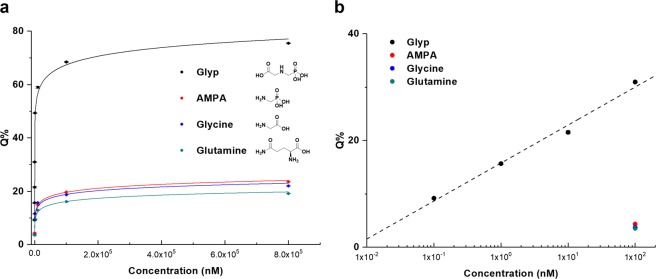
Concentration dependence of the degree of fluorescence quenching (*Q*%) on MIMO-zQ: (**a**) the whole concentration region and (**b**) the low concentration region in the logarithm scale. Each color line shows the estimated fits for different kinds of analyte (the target is glyphosate). The black dotted line indicates the logarithmic linearity between glyphosate and MIMO-zQ. All measurements were performed at five different times.

We applied our recognition system to the detection of glyphosate in aqueous media. Here we investigated concentration-sensitive fluorescence behavior, with the aim of determining the specific binding properties of glyphosate compared to its structural analogues, including aminomethylphosphonic acid (AMPA), glycine and glutamine. Figure 3 shows the fluorescence emissions of glyphosate imprinted (MIMO-zQ’s) and non-imprinted (NIMO-zQ’s) mesoporous organosilica. MIMO-zQ’s exhibited marked sensitive fluorescence quenching upon increased concentrations of glyphosate (*C*_Glyp_) in both graphene QD and InP/ZnS QD, whereas NIMO-zQ’s which have no glyphosate-specific binding sites, exhibited insignificant quenching even in areas of high concentration. This demonstrates that this characteristic of emission quenching clearly denotes the specific binding properties of glyphosate to the imprinted cavity. To test the selectivity potential of this system, we collected fluorescence spectra of MIMO-zQ as a function of each concentration of structural analogues, and the results thus obtained were then quantitatively elucidated by calculating the degree of percentage quenching (*Q*%) (Fig. 4). Even at the lowest concentration of glyphosate (0.1 nM), MIMO-zQ produced a clearly observable *Q*%, 9.2 ± 0.18%. In addition, the PL quenching reaches *ca*. 22% at a glyphosate concentration of 10 nM, while its structural analogues (AMPA, glycine, and glutamine) show the saturation of quenching at a maximum of 24%, upon 800 µM, *ca*. 10^5^ times higher concentration. This is also sensitively correlated to the concentration and shows a good linear relationship with the logarithm of molarity, *log C*_Glyp_, in the region below 1 µM (Fig. 4b). Despite the similarity in the chemical structure and moieties, MIMO-zQ retains a glyphosate-specific recognition property, allowing noticeably sole concentration-sensitive fluorescence quenching properties towards glyphosate as low as a sub-nanomolar level. Different functional silane precursor pairings were applied in the preparation of MIMO to examine the effects of both the charge-charge interaction and the hydrogen bonding between the monomer and imprint molecule during the process of binding site formation. Two trialkoxysilyl precursors bearing propyl-4,5-dihydroimidazole or propyl-*N*,*N*,*N*-trimethylammonium were used to synthesize the control mesoporous silica containing graphene QD and the quenching behavior towards glyphosate of each was then examined (Supplementary Fig. 5). Only subtle fluorescence quenching was observed when compared with MIMO-zQ, indicating that the employed zwitterion played a significant role in the complexation with a target and QD quenching as well. This novel feature, coupled with the molecularly imprinted mesoporous silica and functional imidazolium zwitterion monomers for ultrahigh recognition of glyphosate, may expand the current trend of utilizing fluorescent sensing probes in glyphosate detection.

## Discussion

Although it is difficult to precisely explain the mechanisms involved in fluorescence linear quenching in terms of logarithms of concentration, these findings provide a simple approach to explicating a novel zwitterion modified molecularly imprinted mesoporous organosilica. A zwitterion precursor-glyphosate complex was designed for the development of specific binding cavities which only respond to the target molecules. During the formation of the mesoporous organosilica matrix, we postulate that, the complexes are implanted in the framework of the matrix. The following extraction process detaches only glyphosate exposed to the framework and the counter moiety of the complex is left intact in the vicinity of the resultant cavities. Selectivity enhancement towards glyphosate may be attributed to interaction with the zwitterion functional group as well as the binding site geometrical specificity, which makes a significant difference to the rebinding properties, to differentiate itself from other molecules (AMPA and glycine) which are also sterically capable of fitting into the cavities. Unlike QD colloidal suspension, multiple QDs encapsulated in the matrix have a regular layout in a confined volume. They are placed in the channels of mesopores, in a triangular array and honeycomb-like arrangement. The total initial emission intensity (*F*_0_) of QDs could be expressed as a sum of intensities from effective (*F*_e_) and non-effective (*F*_ne_) ones which are influenced by the MIMO-zQ surroundings and independent from them, for example, QDs when placed deep inside of the matrix, respectively (Equation 1). The population of non-effective QD is expected to be *ca*. 20% of total emissive QDs from the glyphosate rebinding graph (Fig. 4a).

1 $${F}_{0}={F}_{{\rm{e}}}+{F}_{{\rm{ne}}}$$

Three different quenching components may contribute to the quenching process in the presence of analytes, which are from specific binding (*Q*_sb_), non-specific binding (*Q*_nsb_), and non-binding (*Q*_nb_) within cavities by the order of *Q*_sb_ > *Q*_nsb_ > *Q*_nb_.

2 $$F={F}_{0}-({Q}_{{\rm{sb}}}+{Q}_{{\rm{nsb}}}+{Q}_{{\rm{nb}}})$$

where *F* represents the overall emission intensity in the analyte assessment. *Q*% (Equation 3) was analyzed to minimize the unnecessary contribution of *F*_ne_. From the results of NIMO-zQ, the quenching from non-binding results is negligibly small *Q*_nb_, in fact, below 1 µM. Non-specific binding tests with single analyte solutions of the three structural analogues revealed that an average *Q*% of 3.86 ± 0.03% at 100 nM from MIMO-zQ, indicating that the quenching process induced by non-specific binding (*Q*_nsb_) is dominant in the case of these analogues. AMPA and glycine exhibit slightly larger *Q*% than glutamine due to an intimate structural similarity to glyphosate at high concentration. Specific binding quenching, *Q*_sb_, shows a remarkably sensitive change only when it comes to glyphosate, especially at very low concentrations such as below 100 nM. We observed *Q*% of 9.2 ± 0.18% as low as 0.1 nM concentration (0.017 ppb), three times lower than the least detectable dose of commercial ELISA kits (0.05 ppb). Quenching is derived from Förster resonance energy transfer (FRET), which is generally most efficient when the distance between the luminophore and the quencher is 10~60 Å^35^, with the porous matrix having binding cavities and displaying a linearity against *log C*_Glyp_, while a similar QDs-incapsulated bisphenol-A imprinted mesoporous organosilica system exhibits an almost linear relationship with analyte concentration. It retains only flimsy binding hollows derived from the cleavage of sacrificial urethane bond without any rebinding functional group^30^. This highly sensitive and specific response towards the target in MIMO-zQ could not be explained by the employment of conventional models. Hence, in spite of the clear necessity of further and more fundamental studies, we could only hypothesize that the zwitterionic group plays an additional role in the enhancement of FRET with multiple QDs placed nearby channels *via* the matrix. FRET is presumably regulated by the extent of the electronic state change in the charge separated zwitterionic group by analyte species, which may imply a certain strength of interaction by complex formation. It results in the same quenching trends regardless of the species of QDs employed. Alternations to the zwitterion functional group to ammonium or dihydroimidazole group resulted in a failure to produce a sensitive fluorescent probe, presumably owing to their weaker FRET quenching abilities when compared to zwitterions, as well as the insufficient interaction with glyphosate, which prevents formation of the precursor-glyphosate complex and binding cavities (Supplementary Fig. 5).

In order to exclude the influence of chelate formation by metal ions^36^, the analytes dissolved in pure de-ionized water were tested without the use of any buffer solutions. In the un-degassed de-ionized water, the measured pH values remained at *ca*. 6.86 in the concentration range from 0.1 nM to 100 nM. The most probable proton dissociated species of polyprotic acids were calculated using the corresponding pH and computed p*K*_a_ values (Supplementary Table 1), and the results indicate that the formal charges of glyphosate, AMPA, glycine and glutamine are −2 (93.6%), −1 (90.1%), 0 (99.8%) and 0 (99.4%), respectively (Supplementary Table 2). Whilst further studies are clearly needed into the effects of negative formal charges of target molecules during the molecular recognition process when aided by cavity geometry, we are able to confirm that our probe presents a stable performance towards the target analyte.

## Conclusions

In summary, this paper details the development of an approach and design principle for fluorescent glyphosate probes which may be of interest to other researchers, achieving at the same time, an improvement of selectivity in combination with ultrahigh sensitivity. The simple incorporation of a precursor containing an imprint molecule-binding imidazolium zwitterionic functional group, led to a noticeable level of progress in MIMO applications. This in turn, opens up not only the potentially more widespread applications of a nanoporous imprinting matrix but also the potential for the imprinting of a precursor-target complex. The electrostatic interaction and hydrogen bonding present in the complex form of the zwitterion monomer and target analyte provide a suitable environment for the formation of homogenous binding sites on the porous matrix, enhancing the fluorescence quenching process at sub-nanomolar levels. Significant fluorescence quenching was even observed to have dropped as low as 0.1 nM, 60 times lower than the maximum level of contaminants limits of the EU (0.1 µg/L, 6 nM) and 40000 times lower than that of US regulations (700 µg/L, 4.1 µM)^37^, allowed by the first report of sensing materials. It is clear that more comprehensive fundamental studies, involving both theoretical calculations, and real sample analyses will be required to fully elucidate the recognition properties at work here. Nevertheless, this molecular imprinting probe design exhibits genuine potential and versatility, and the sensitivity and selectivity displayed here presents distinct advantages over conventional sensing methods. We believe this work represents a clear contribution to our knowledge of the conventional pathways of synthesis in MIMO, as well as providing significant guidance for the future practical applications of these processes.

## Methods

### Characterization

Solid-state ^13^C NMR spectra were recorded on a Agilent 400/54 Premium Shielded NMR spectrometer equipped with a CP/MAS probe. High resolution transmission electron microscopy (HRTEM) images were obtained by means of a JEOL JEM 2100 operating at 200 kV. FT-IR measurements were carried out using Thermo scientific NICOLET iS10. Brunauer-Emmet-Teller (BET) surface area analysis was conducted using a Belsorp-Max (BEL Japan. Inc.) analyzer. Pore size distribution was obtained using the Barret-Joyner-Halenda (BJH) model on the adsorption branch. Small angle X-ray scattering was analyzed with an Anton Paar SAXSess (Cu Kα radiation, λ = 1.54 Å). Fluorescence spectra measurements were performed with the use of a Perkin Elmer LS 45 luminescence spectrometer equipped with a 10 mm quartz emission cuvette from Helma.

### Synthesis of the zwitterionic organicsilica precursor of 3-(4-sulfonatobutyl)-1-[3-(triethoxysilyl)propyl]-1H-imidazol-3-ium (z)

z was synthesized according to a protocol adapted from Miao *et. al*.^38^. Sodium ethanolate (6.8 g, 95%, EMD Millipore) was dissolved under N_2_ to an absolute ethanol solution (100 mL) of imidazole (6.8 g, 99%, Sigma Aldrich). The mixture was then stirred at 70 °C for 8 h. 3-Chloropropyl(triethoxy)silane (24.08 g, 98%, Sigma Aldrich) was added dropwise, and the mixture was refluxed for 12 h under N_2_. The solution was then filtered to remove any byproducts, and the solvent was removed by rotatory evaporation, yielding the product (*N*-(3-propyltriethoxysilane)imidazole) as a viscous light-yellow liquid. Following this, the as-synthesized product (10 g) and oxathiane 2,2-dioxide (5 g, 99%, Sigma Aldrich) were mixed in ethanol (70 mL) and stirred at 50 °C for 48 h. The solution was washed with diethyl ether several times and dried in a vacuum oven at 90 °C to remove any remaining solvents and byproducts. A yellow-colored wax was obtained with a yield of 80%.

### Synthesis of glyphosate-imprinted mesoporous organosilica nanoparticles (MIMO-z)

An aqueous NaOH solution (1.0 M, 7.0 mL, Sigma Aldrich) was added to a solution of hexadecyl(trimethyl)azanium bromide (CTAB, 0.94 g, 99%, Sigma Aldrich) in de-ionized water (480 mL). After adding the mixed solution of 2-(phosphonomethylamino)acetic acid (glyphosate, 50 mg, 96%, Sigma Aldrich) and **z** (70 mg) dissolved in distilled water, tetraethyl silicate (TEOS, 5.12 g, 99%, Sigma Aldrich) was added dropwise. The reaction mixture was subsequently stirred at 70 °C for 3 h. The precipitated product was filtered, washed with distilled water, ethanol and acetone, and dried in a vacuum oven at 60 °C for 2 days. The resultant product (2.0 g) was refluxed in a mixed solution of HCl (35 wt%, 10 g), distilled water (50 g) and 1,4-dioxane (150 g) at 110 °C for 24 h. The product (MIMO-z) was then isolated by filtration, washed with distilled water and acetone, and dried in *vacuo* at 60 °C for 3 days. Non-imprinted mesoporous organosilica (NIMO-z) nanoparticles were prepared in the same way except that no glyphosate was added. Both propyl-4,5-dihydroimidazole or propyl-*N*,*N*,*N*-trimethylammonium moiety functionalized MIMO were synthesized using 3-(4,5-dihydroimidazol-1-yl)propyl-triethoxysilane (97%, Gelest, Inc.) or trimethyl(3-trimethoxysilylpropyl)azanium chloride (97%, Sigma Aldrich) instead of **z**, respectively.

### Synthesis of the thiol group-functionalized glyphosate imprinted mesoporous organosilica nanoparticles

MIMO-z (1.0 g) was dispersed in anhydrous toluene (100 mL). 11-trimethoxysilylundecane-1-thiol (100 mg, 95%, Gelest, Inc) was added dropwise at 80 °C and the reaction mixture was stirred at 100 °C for 24 h. The product was filtered, washed with ethanol and acetone, and dried in *vacuo* at 70 °C for 24 h.

### Synthesis of the graphene or InP/ZnS quantum dot-encapsulated glyphosate imprinted mesoporous organosilica nanoparticles (MIMO-zQ’s)

Graphene quantum dots (5 mL, 1 mg mL^−1^ in H_2_O, Sigma Aldrich) and InP/ZnS quantum dots (1 mL, 5 mg mL^−1^ in toluene, Sigma Aldrich) were added to a suspension of thiol group-functionalized MIMO-z (500 mg) in H_2_O and toluene, respectively. The mixture was stirred for 6 h and isolated by filtration, washed with distilled water or toluene and dried in *vacuo* at 30 °C for 5 days. NIMO-zQ’s nanoparticles were prepared in the same way except for the use of NIMO-z instead of MIMO-z.

### Glyphosate detection

All of the analytical solutions of glyphosate and its analogues were prepared by dissolving them in de-ionized water. The diluted MIMO-zQ/NIMO-zQ suspension (2 mg mL^−1^) was prepared using ethanol. In assessing both sensitivity and single-component selectivity, MIMO-zQ/NIMO-zQ suspension (50 µL) and different concentration of analytes (2.0 mL) were mixed and stirred in each vial for 5 mins, and the fluorescence was then measured at 350 nm excitation wavelength. The intensities of emission were recorded at λ_max_ of InP/ZnS QD (532 nm) and graphene QD (451 nm). In order to assess the intensity change from *F*_*e*_, equation (1), the degree of fluorescence quenching (*Q*%) was calculated using the following equation,

3 $$Q \% =({F}_{0}-F)/{F}_{0}\times 100$$

where *F*_0_ and *F* are intensities at λ_max_ in the absence and presence of a specific concentration of the analyte, respectively. All of the analytes were used without any additional purification.

## Supplementary information

Supporting Information
