# Supplementary material for: Advanced method for fabrication of molecularly imprinted mesoporous organosilica with highly sensitive and selective recognition of glyphosate
Source: Sci Rep. 2019 Jul 16;9:10293. doi: 10.1038/s41598-019-46881-7 (PMC6635376; doi:10.1038/s41598-019-46881-7)
Supplement: Supplementary file 1 — Supporting Information [file 41598_2019_46881_MOESM1_ESM.docx]

Supplementary Information

Advanced method for fabrication of molecularly imprinted mesoporous organosilica with highly sensitive and selective recognition of glyphosate

Youngdo Kim,^1*^ Jaeho Lee^1*^ and Ik-Soo Shin^2^

^1^Biosensor and Materials Group, Korea Institute of Science and Technology Europe, Universität des Saarlandes Campus E7 1, 66123 Saarbrücken, Germany

^2^Department of Chemistry, College of Natural Science, Soongsil University, Seoul 06978, Republic of Korea

*Corresponding authors, Dr. Youngdo Kim, yd.kim@kist-europe.de; Dr. Jaeho Lee, jaeho.lee@kist-europe.de


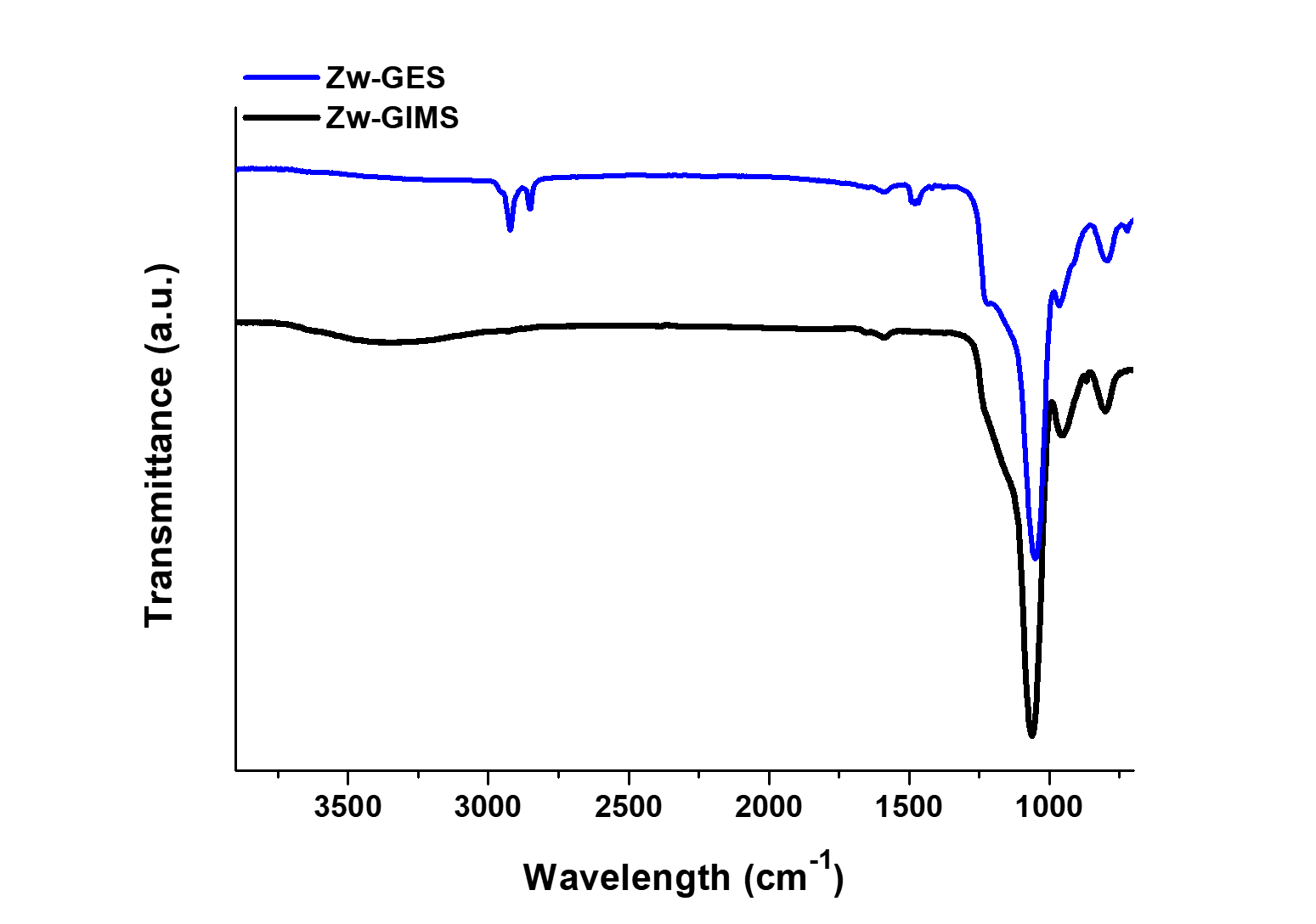


**Supplementary Figure 1:** FT-IR spectra of as-synthesized organosilica before (blue line) and after (MIMO-z; black line) extraction process.

**Supplementary Note 1: Porous Structure Analysis - specific surface area, pore size distribution measurement, and ordered mesopore array**

Investigations of MIMO-z series using N_2_ adsorption/desorption isotherms revealed a porous nature (Supplementary Figure 2a,b). Using the Brunauer-Emmett-Teller (BET) surface area as a comparable trait, both MIMO-z and MIMO-zQ exhibited type IV curves which are characteristic of a mesoporous structure^1^, whereas the QD encapsulated ones were found to have a relatively lower BET surface area (365 m^2^ g^-1^) compared to MIMO-z (479 m^2^ g^-1^). We next measured pore size distribution by means of the Barrett-Joyner-Halenda (BJH) method. Both exhibited average pore sizes that were slightly different (2.0 and 1.7 nm). The decrease in the BET surface area and the average pore size of the QD encapsulated ones was due to the incorporation of QD particles onto the pore surface of MIMO-z, resulting in the occupation of mesopores and the decrease in surface area and pore size. This further proved that the QD had been grafted onto the pore surface of MIMO-z. Small angle X-ray Scattering (SAXS) analyses also confirmed the structure of ordered mesopores; three characteristic peaks that are assigned as (100), (110) and (200) reflections are correlated to the 2D hexagonal (P6mm) structure^2^ (Supplementary Figure 2c,d). Considering each lattice parameter and average pore size, the wall thickness among pores could be estimated, which was approximately 4.1 nm^3^. Indeed, it suggests that both zwitterionic monomer and glyphosate could make a stable complex, and the imprinted site is well developed on the silica framework among pores.


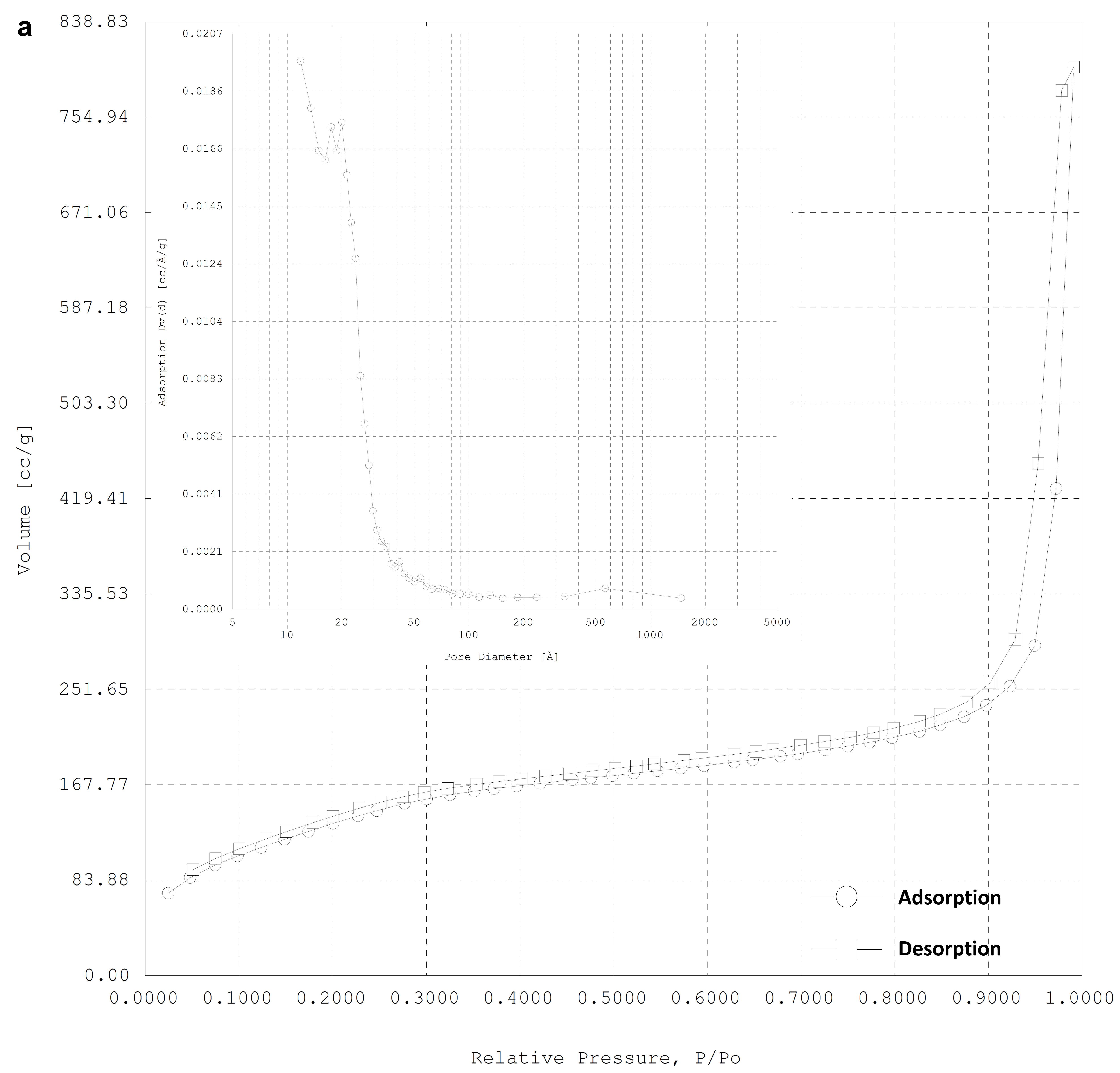


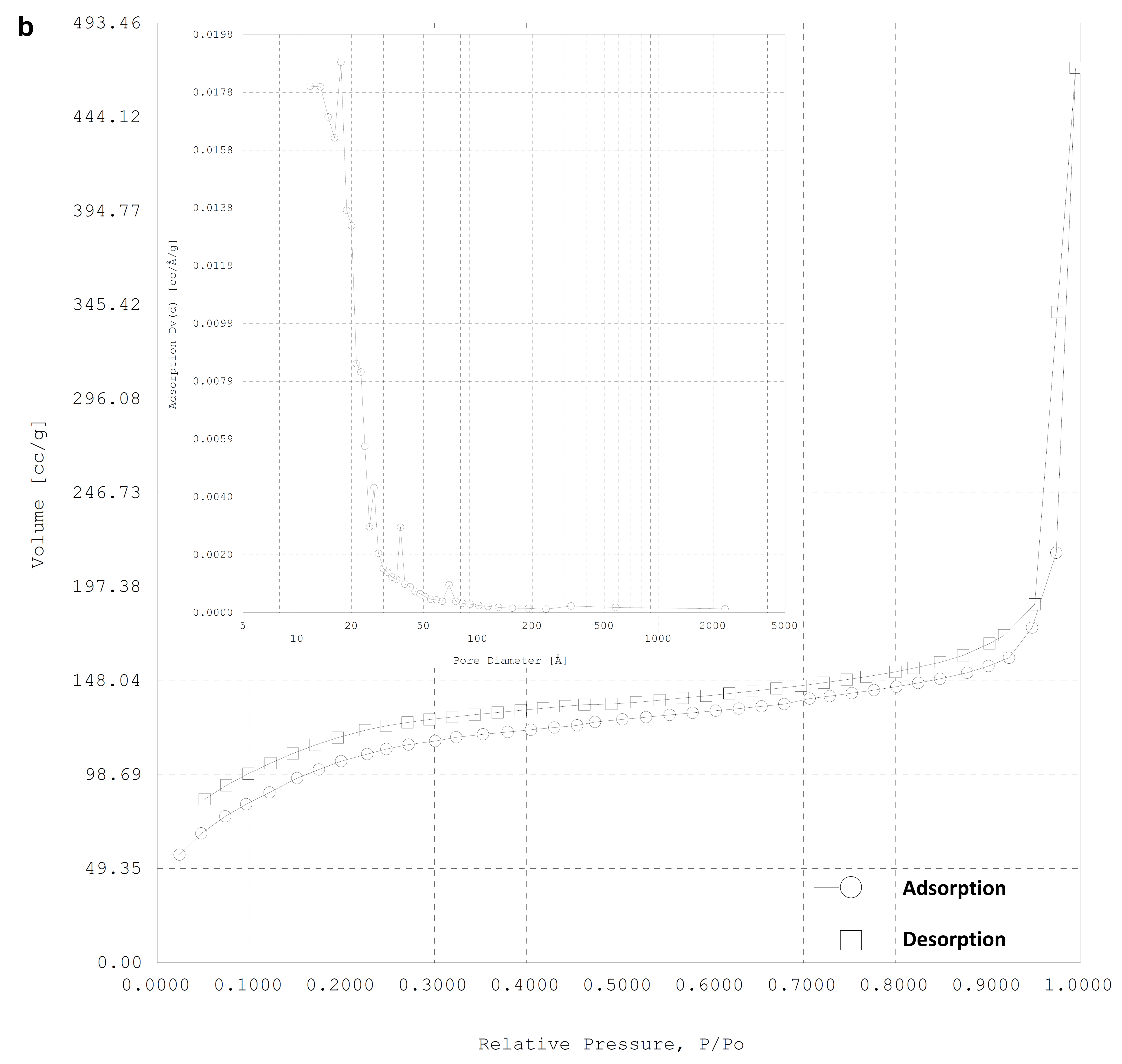


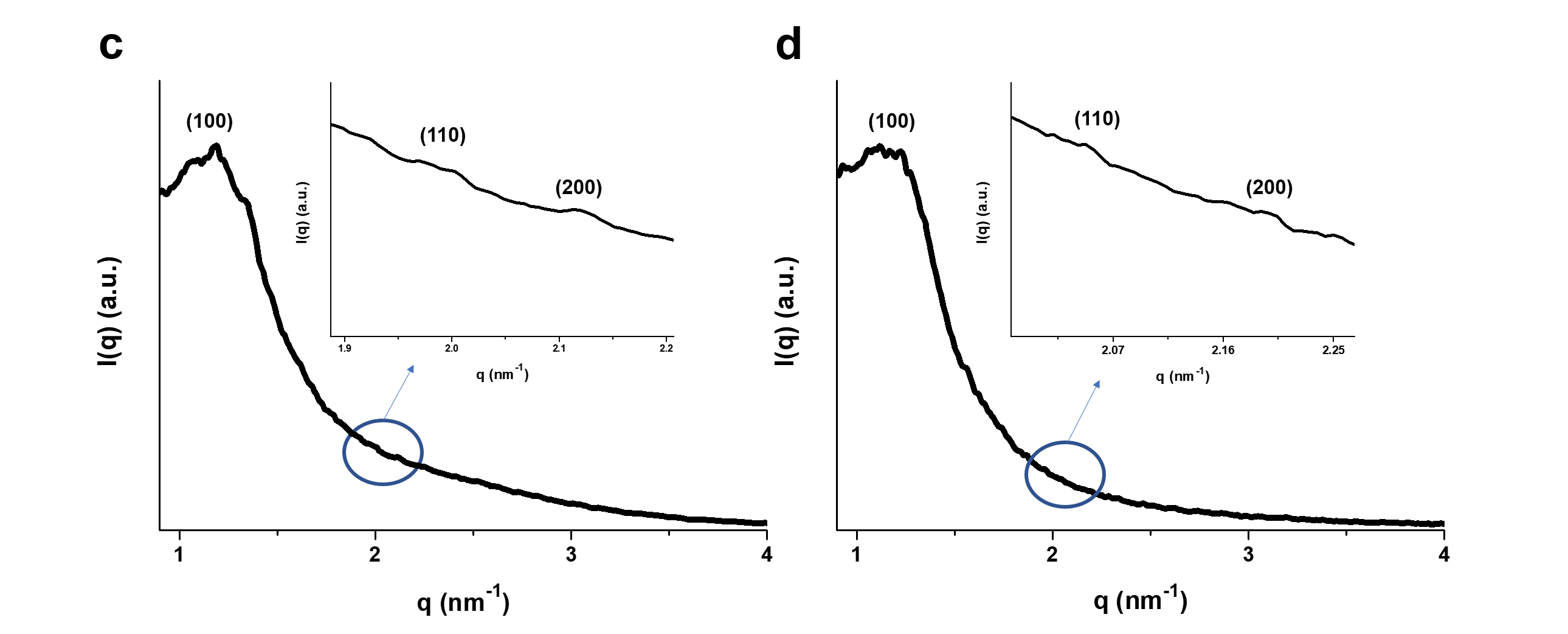


**Supplementary Figure 2:** Porous structure analysis of mesoporous organosilica. **a,b** Nitrogen adsorption-desorption isotherm and BJH pore size distribution curve for MIMO-z and MIMO-zQ, respectively. **c, d** Small angle X-ray scattering curve of MIMO-z and MIMO-zQ, respectively.

**
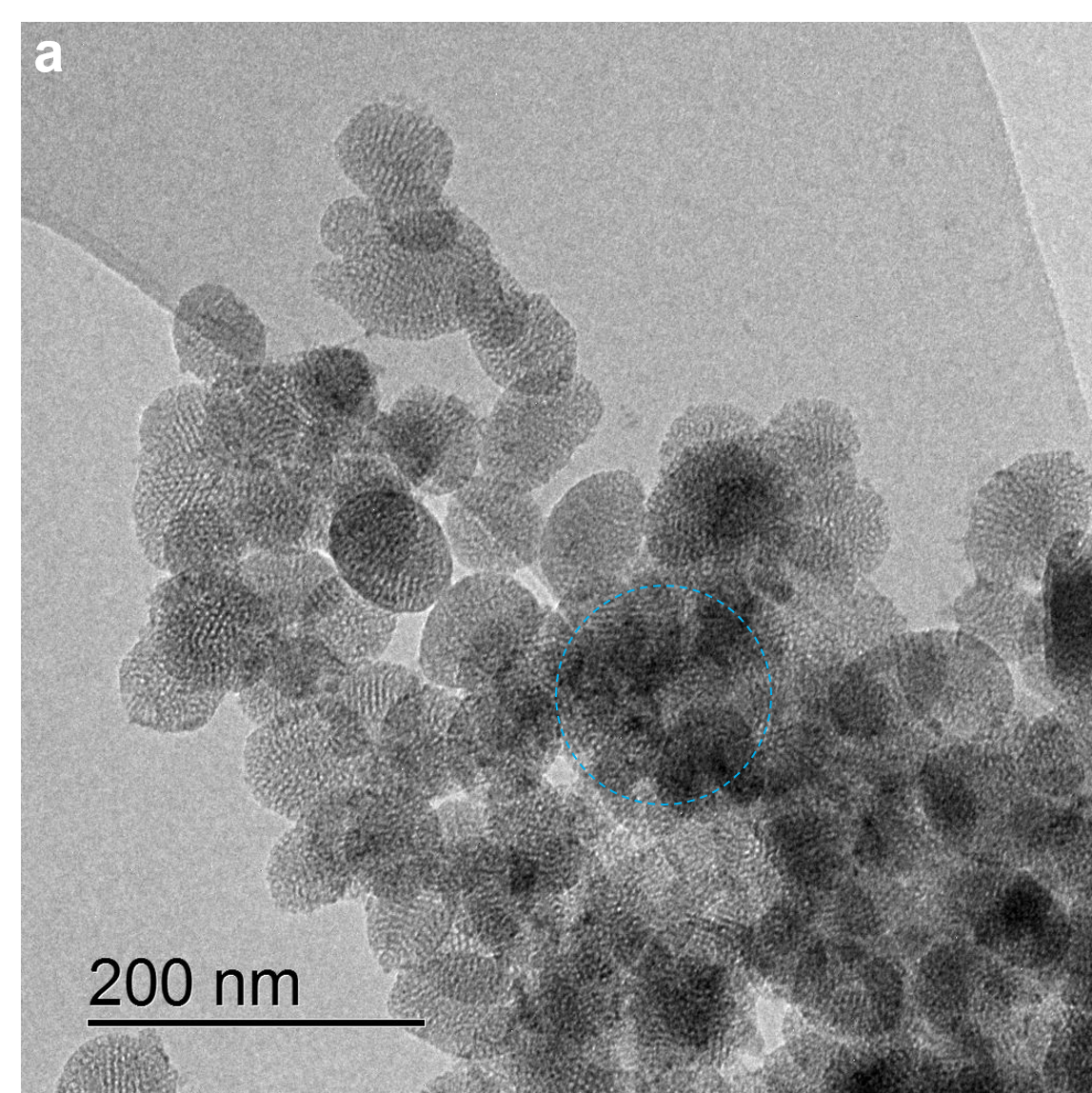
**

**
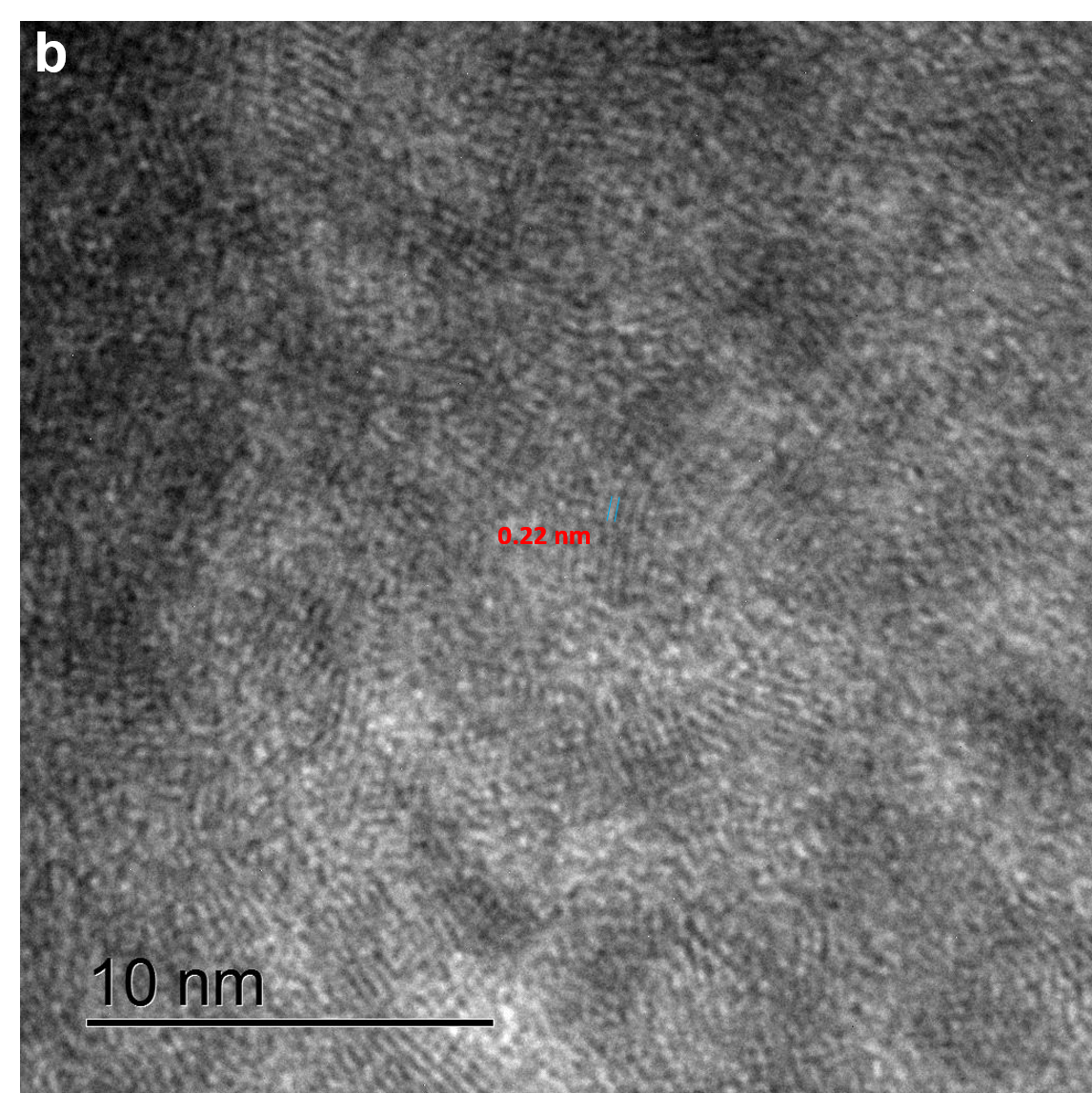
**

**Supplementary Figure 3:** HRTEM micrographs of mesoporous organosilica. **a** Image of InP/ZnS QD encapsulated MIMO-z. **b** Enlarged image of a part (blue dot circle) of Fig. S3a.

**Supplementary Note 2: Solid state ^13^C NMR**

MIMO-z was analyzed by solid state ^13^C CP/MAS NMR spectroscopy (Supplementary Figure 4). This revealed methylene carbons of the thiol-functionalized hydrophobic groups^4^ which showed a strong signal at around 25 ppm. It also retained the imidazolium group^5^ which exhibited broad peaks observed at around 137-120 ppm. These signals, corresponding to the propyl carbons^4,6,7^ bonded to Si appeared at around 6, 12 and 45 ppm.


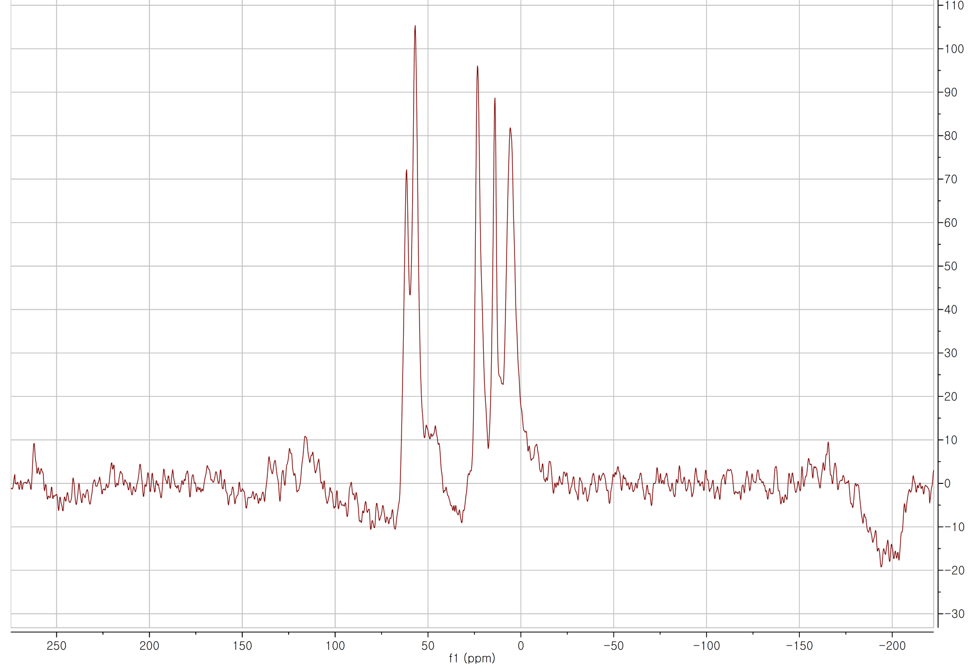


**Supplementary Figure 4:** ^13^C CP/MAS solid state NMR spectroscopy of MIMO-z.


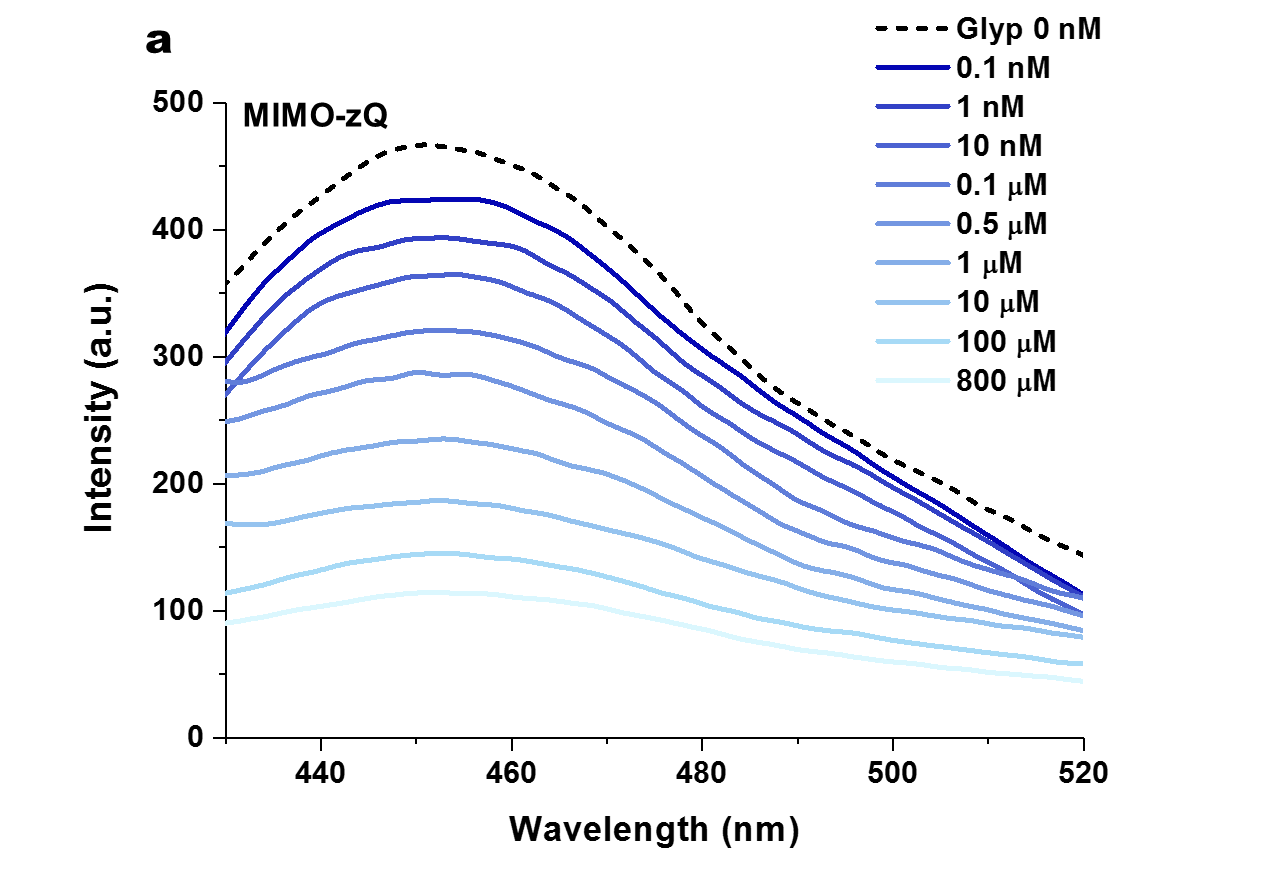

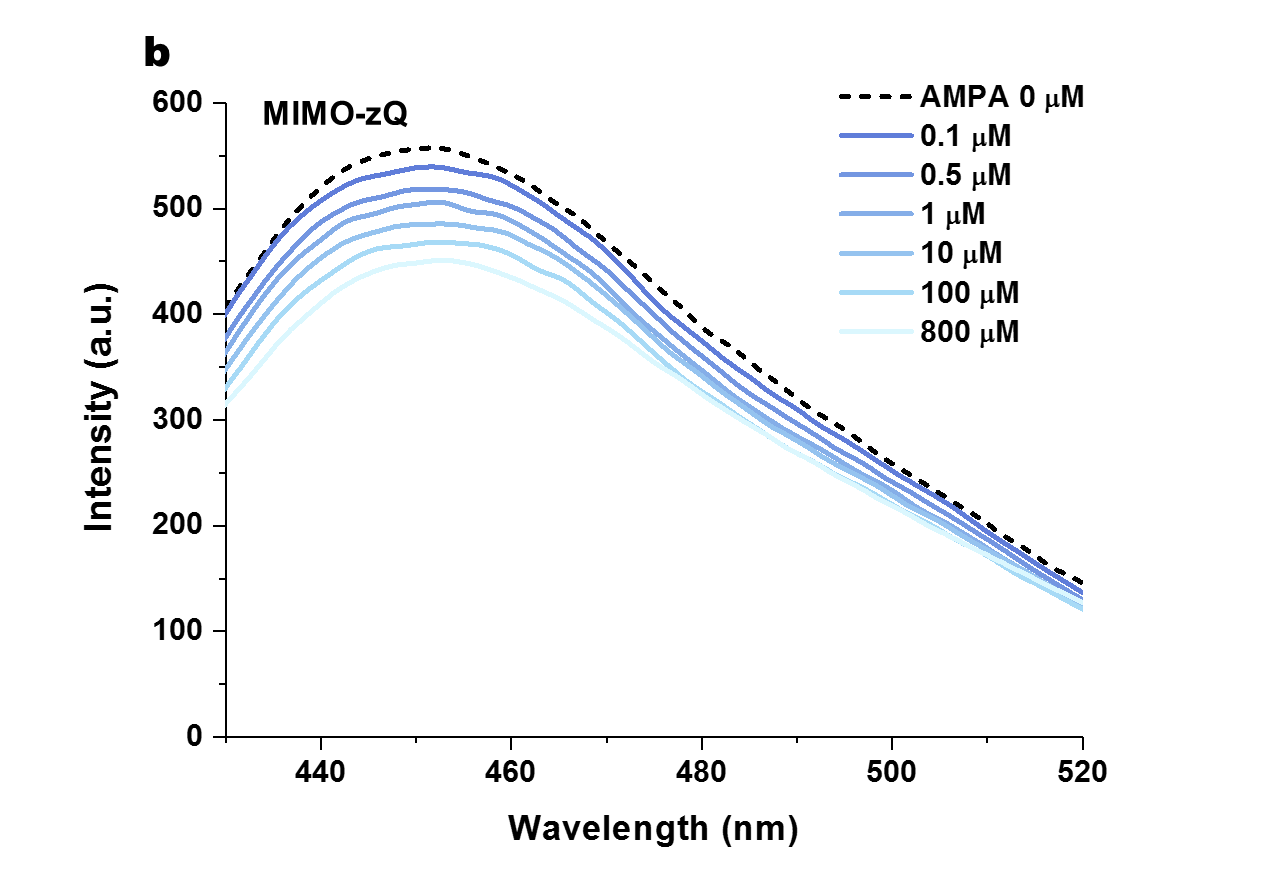


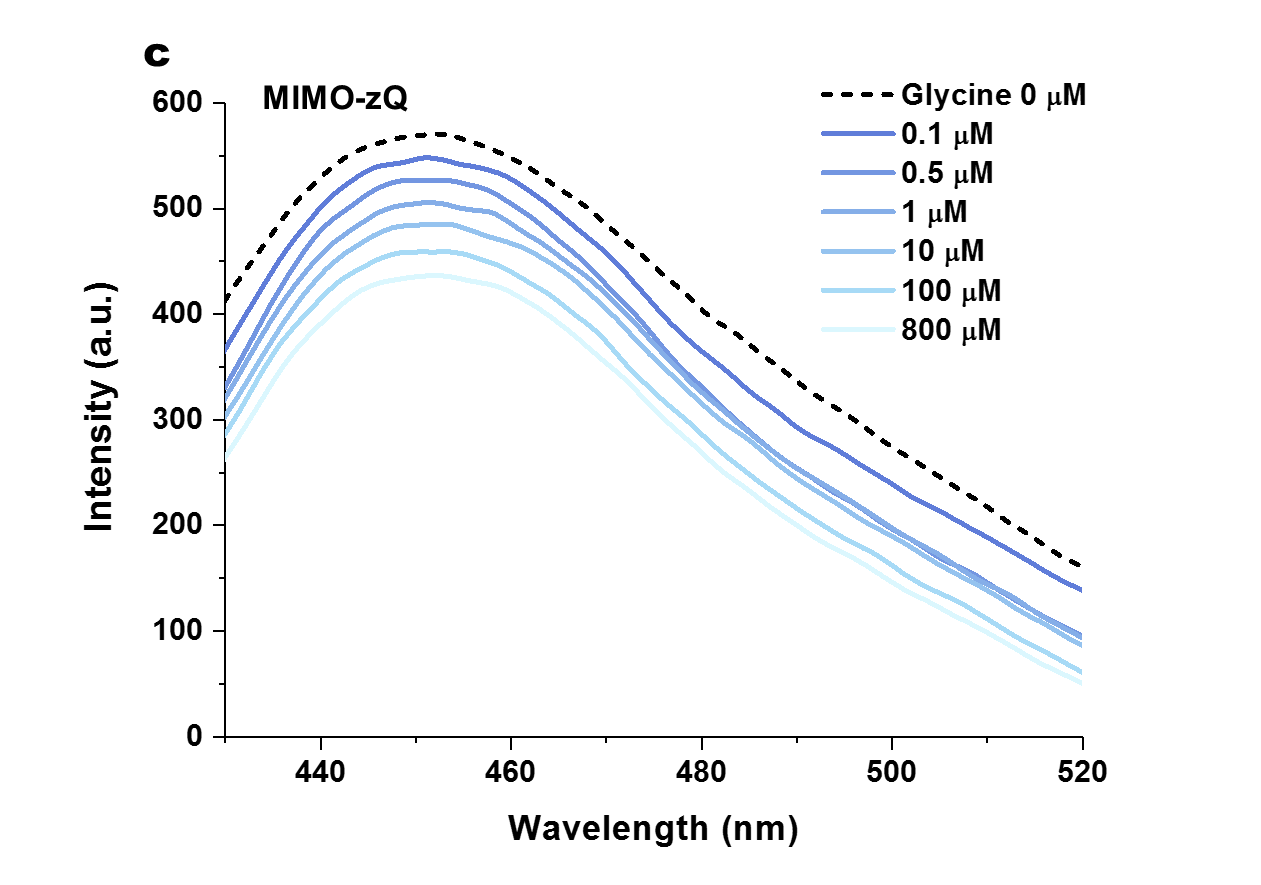

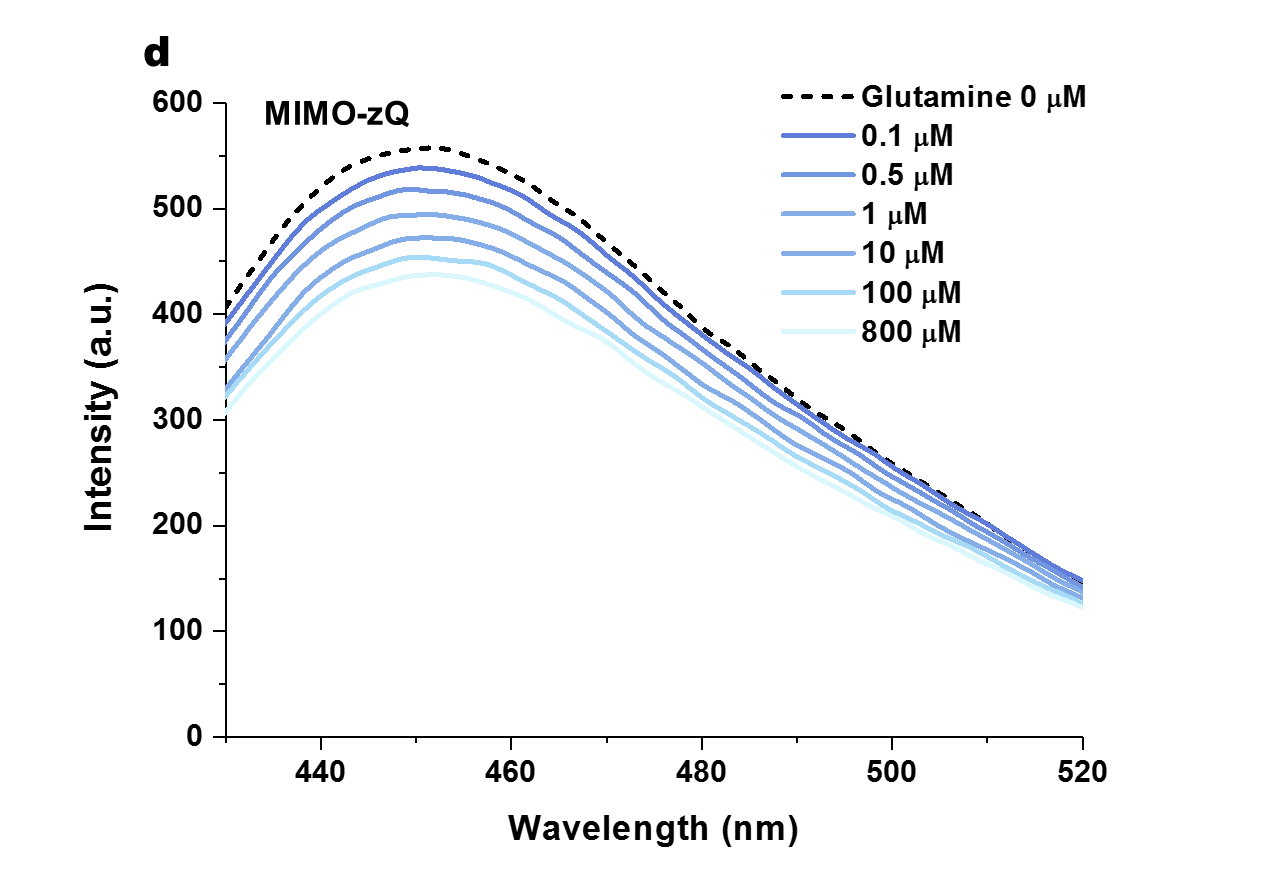


**Supplementary Figure 4:** Photoluminescence(PL) emission change as function of analyte concentration for selectivity tests: **a**. glyphosate; **b**. aminomethylphosphonic acid (AMPA); **c**. glycine; **d**. glutamine


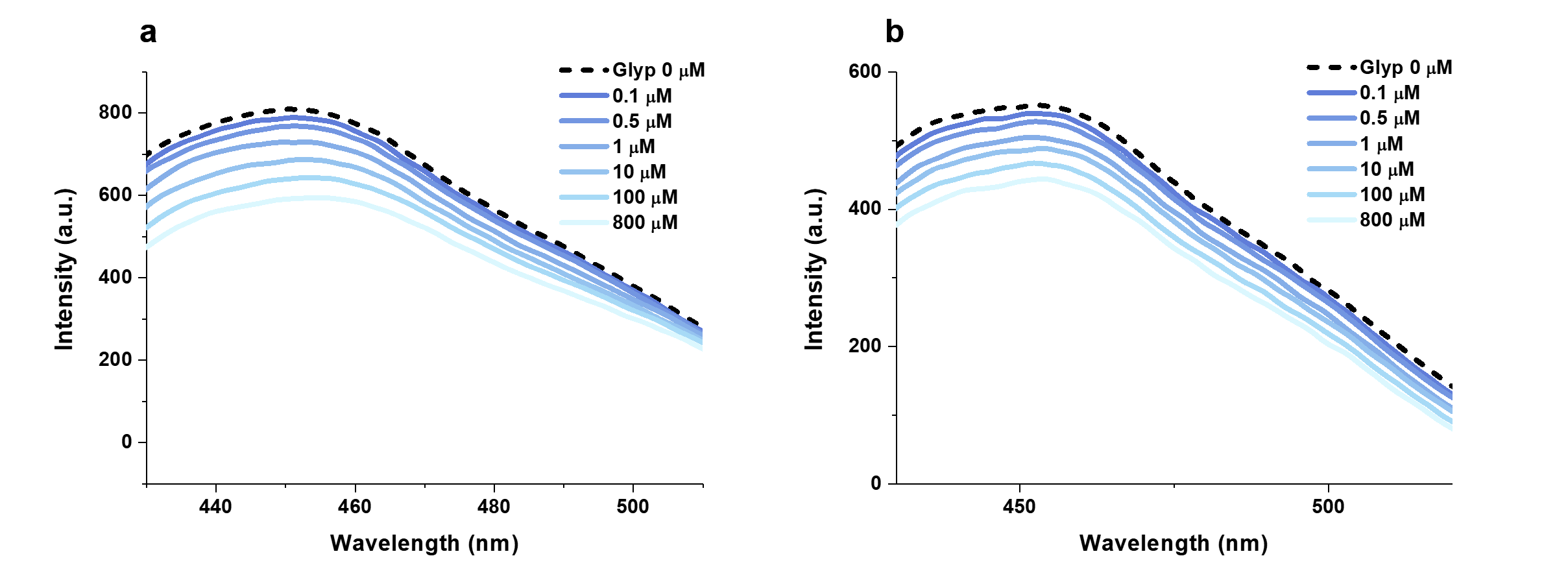


**Supplementary Figure 5:** Fluorescence quenching profiles of different functional group precursor involved MIMO-Q’s: **a**. propyl-4,5-dihydroimidazole and **b**. propyl-*N,N,N*-trimethylammonium obtained with increasing glyphosate concentration.

**Supplementary Note 3: Calculation of fractional dissociation for glyphosate, glycine, glutamine, and AMPA**

The fractional concentrations of proton dissociated species for each compound were calculated with well-known equations for equilibrium fractional concentration, *α*, of triprotic acids (Supplementary Equation 1~4) and diprotic acids (Supplementary Equation 5~7). All the stepwise proton dissociated species and the equilibrium constants were illustrated in Supplementary Scheme 1 and Table 1, respectively.

1. Glyphosate


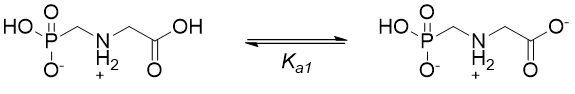


H_3_T H_2_T^-^


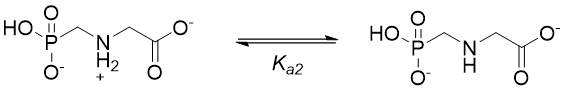


H_2_T^-^ HT^2-^


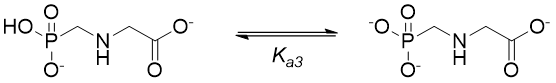


HT^2-^ T^3-^

1. Amino acids: R = H- (glycine), H_2_NCOCH_2_CH_2_- (glutamine)


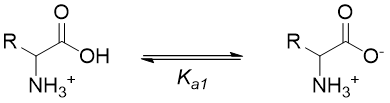


H_2_D^+^ HD


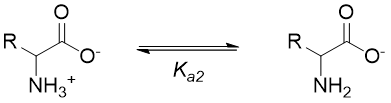


HD D^-^

1. Aminomethylphosphonic acid (AMPA)


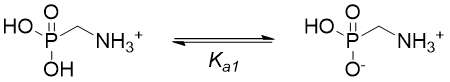


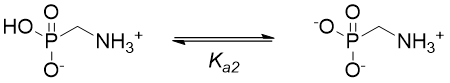


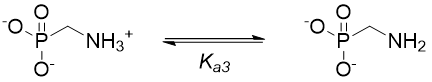


**Supplementary Scheme 1:** Stepwise deprotonation reactions of glyphosate (a), glycine and glutamine (b) and AMPA (c) used in the fractional concentration calculation.

$\alpha_{H_{3}T}=\frac{{[H^{+}]}^{3}}{{[H^{+}]}^{3}+{K_{a1}[H^{+}]}^{2}+K_{a1}K_{a2}\left[ H^{+} \right]+K_{a1}K_{a2}K_{a3}}$ (1)

$\alpha_{H_{2}T^{-}}=\frac{K_{a1}{[H^{+}]}^{2}}{{[H^{+}]}^{3}+{K_{a1}[H^{+}]}^{2}+K_{a1}K_{a2}\left[ H^{+} \right]+K_{a1}K_{a2}K_{a3}}$ (2)

$\alpha_{HT^{2-}}=\frac{K_{a1}K_{a2}\left[ H^{+} \right]}{{[H^{+}]}^{3}+{K_{a1}[H^{+}]}^{2}+K_{a1}K_{a2}\left[ H^{+} \right]+K_{a1}K_{a2}K_{a3}}$ (3)

$\alpha_{T^{3-}}=\frac{K_{a1}K_{a2}K_{a3}}{{[H^{+}]}^{3}+{K_{a1}[H^{+}]}^{2}+K_{a1}K_{a2}\left[ H^{+} \right]+K_{a1}K_{a2}K_{a3}}$ (4)

$\alpha_{{H_{2}D}^{+}}=\frac{{[H^{+}]}^{2}}{{[H^{+}]}^{2}+K_{a1}[H^{+}]+K_{a1}K_{a2}}$ (5)

$\alpha_{HD}=\frac{K_{a1}[H^{+}]}{{[H^{+}]}^{2}+K_{a1}[H^{+}]+K_{a1}K_{a2}}$ (6)

$\alpha_{D^{-}}=\frac{K_{a1}K_{a2}}{{[H^{+}]}^{2}+K_{a1}[H^{+}]+K_{a1}K_{a2}}$ (7)

All the analyte dissolved in unbuffered D.I. water under the concentration range from 0.1 nM to 100 nM have pH at *ca*. 6.86 and the fractional concentration of each chemicals were calculated with each measured value (Supplementary Table 1 and 2).

**Supplementary Table 1:** p*K_a_* values of the compounds.

| Compound | p*K_a_* | | | Ref. |
| --- | --- | --- | --- | --- |
|  | p*K_a1_* | p*K_a2_* | p*K_a3_* |  |
| Glyphosate | 0.47 | 5.69 | 11.81 | [8] |
| Glycine | 2.34 | 9.60 |  | [9] |
| Glutamine | 2.17 | 9.13 |  | [9] |
| AMPA | 2.35 | 5.9 | 10.8 | [10] |

**Supplementary Table 2:** Calculated fractional concentration of proton dissociated species of tested chemicals at pH = 6.86.

| Compound |  | | Formal charge | | |  | |
| --- | --- | --- | --- | --- | --- | --- | --- |
|  | +1 | 0 | | -1 | -2 | | -3 |
| Glyphosate |  | 2.58×10^-8^ | | 6.33×10^-2^ | 0.936 | | 1.05×10^-5^ |
| Glycine | 3.01×10^-5^ | 0.998 | | 1.82×10^-3^ |  | |  |
| Glutamine | 2.03×10^-5^ | 0.994 | | 5.34×10^-3^ |  | |  |
| AMPA | 3.05×10^-6^ | 9.88×10^-2^ | | 0.901 | 1.03×10^-4^ | |  |

|  |  |  |
| --- | --- | --- |

**Supplementary References**

1. Huh, S., Wiench, J. W., Yoo, J.-C., Pruski, M. & Lin, V. S.-Y. Organic functionalization and morphology control of mesoporous silicas via a co-condensation synthesis method. *Chem. Mater.* **15**, 4247-4256 (2003).

2. Choi, M., Heo, W., Kleitz, F. & Ryoo, R. Facile synthesis of high quality mesoporous SBA-15 with enhanced control of the porous network connectivity and wall thickness. *Chem. Commun.* **0**, 1340-1341 (2003).

3. Zhao, D., Feng, J., Huo, Q., Melosh, N., Fredrickson, G. H., Chmelka, B. F. & Stucky, G. D. Triblock copolymer syntheses of mesoporous silica with periodic 50 to 300 angstrom pores. *Science* **279**, 548-552 (1998).

4. Ki, C. D., Emrick, T. & Chang, J. Y. Preparation of functional nanoporous silica for encapsulation of CdSe nanoparticles. *Adv. Mater.* **17**, 230-233 (2005).

5. Litschauer, M. & Neouze, M.-A. Nanoparticles connected through an ionic liquid-like network. *J. Mater. Chem.* **18**, 640-646 (2008).

6. Jung, B. M., Kim, M. S., Kim, W. J. & Chang, J. Y. Molecularly imprinted mesoporous silica particles showing a rapid kinetic binding. *Chem. Commun.* **46**, 3699-3701 (2010).

7. Kim, Y., Lee, K. M. & Chang, J. Y. Highly luminescent tetra(biphenyl-4-yl)ethene-grafted molecularly imprinted mesoporous silica nanoparticles for fluorescent sensing of diethylstilbestrol. *Sens. Actuators B-Chem.* **242**, 1296-1304 (2017).

8. Peixoto, M. M., Bauerfeldt, G. F., Herbst, M. H., Pereira, M. S. & da Silva, C. O. Study of the Stepwise Deprotonation Reactions of Glyphosate and the Corresponding p*K*_a_ Values in Aqueous Solution. *J. Phys. Chem. A* **119,** 5241–5249 (2015).

9. Budavari, S., ed. *The Merck Index: An Encyclopedia of Chemicals, Drugs, and Biologicals*, 12th ed. New Jersey: Merck, 1996.

10. Freedman, L. D. & Doak, G. O. The Preparation and Properties of Phosphonic Acids. *Chem. Rev.* **57,** 479–523 (1957).
